# Unraveling the intricate connection between dietary factors and the success in long-term space missions

**DOI:** 10.1038/s41526-023-00331-x

**Published:** 2023-12-13

**Authors:** Paola Pittia, Stéphane Blanc, Martina Heer

**Affiliations:** 1 University of Teramo, Teramo, Italy; 2 CNRS, Strasbourg, France; 3 IU International University of Applied Sciences, Erfurt, Germany; 4 University of Bonn, Institute of Nutritional and Food Sciences, Bonn, Germany

**Keywords:** Physiology, Nutrition

## Abstract

In recent decades of spaceflight, inadequate caloric intake has posed significant nutritional challenges, contributing to muscle degradation, weakened immune and cardiovascular systems during and after space missions. This challenge becomes more acute on longer exploration missions, where transporting all food for the entire mission becomes a logistical challenge. This places immense pressure on the food system, requiring energy-dense, varied, stable, and palatable food options. Prolonged storage can lead to nutrient degradation, reducing their bioavailability and bioaccessibility to astronauts. Research is essential not only to improve the quality and stability of space food but also to enhance nutrient bioavailability, thereby reducing weight and volume of food. Muscle and bone loss represent major risks during extended spaceflight, prompting extensive efforts to find exercise countermeasures. However, increased exercise requires additional energy intake, and finding the optimal balance between energy needs and the preservation of muscle and bone mass is challenging. Currently, there is no reliable way to measure total energy expenditure and activity-related energy expenditures in real-time. Systematic research is necessary to develop onboard technology for accurate energy expenditure and body composition monitoring. This research should aim to establish an optimal exercise regimen that balances energy requirements while maintaining astronaut strength and minimizing food transport. In summary, this overview outlines key actions needed for future exploration missions to maintain body mass and physical strength of space travellers. It addresses the requirements for food processing and preservation, considerations for space food formulation and production, and the essential measures to be implemented.

## Introduction

**Fig. 1:**
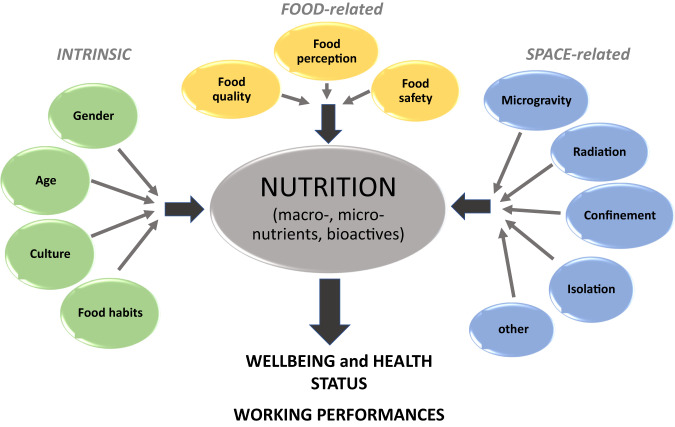
Nutritional intake and wellbeing. Intrinsic, food and space related factors affect nutritional intake and consequently wellbeing, health status and working performances.

Every space traveller is affected by external factors on their way through the Universe. Aside from microgravity per se, radiation, confinement and isolation, nutrition, etc. affect the optimal functioning of the body. ‘Nutrition’ here is defined as the quality and quantity of food to be consumed affecting the nutritional status and all related aspects of optimal physiological performance and wellbeing as depicted in Fig. 1.

**Fig. 2:**
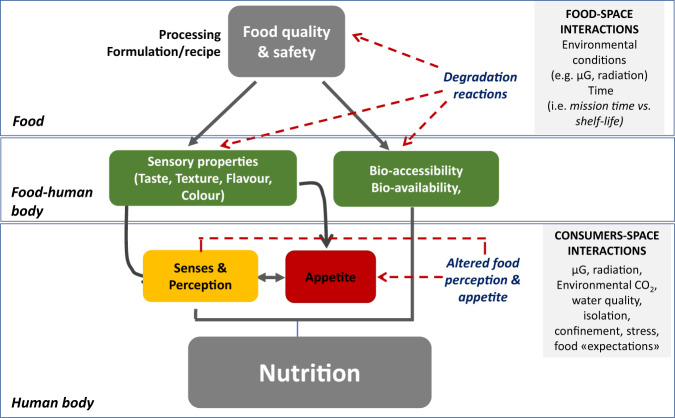
Microgravity, food and consumer. Relation of food and the human body considering, in particular, food-space and consumer-space interactions.

In general, food habits, gender, culture and age impact food selection of everybody, independent of the gravitational environment. During exploration missions, environmental/extrinsic factors (e.g. microgravity, radiation, confinement, isolation, circadian rhythms) and food-related factors (e.g. type, quality, safety) can affect, beside others, food perception and appetite regulation (reviewed in refs. ^1,2^). For instance, considering the lock-down during the COVID-19 pandemic as an example for confinement and reduced physical activity, some studies found increased consumption of ultra-processed food, increased snacking and meal frequency and reduced protein intake^3,4^. Therefore, these factors may play a key role in the adequate supply of both essential nutrients, fluids and other bioactive compounds during missions. Within the impact factors one might distinguish between the “food-related” ones, affecting nutrient/fluid supply and respective interactions, and those related to external or environmental factors such as microgravity, radiation, confinement and isolation (Fig. 2).

This review will focus mainly on food-related aspects such as food quality and safety, sensory properties affecting senses, perception and appetite but also bioavailability and bioaccessibility of nutrients which, as a consequence, will affect nutrient supply and space travellers’ health during long-duration exploration missions.

The main goal of this brief summary is to outline the essential upcoming tasks in future space exploration missions, aiming to maintain body mass and physical strength of astronauts. Additionally, it explores the requirements for food processing and preservation, discusses the considerations involved in formulating and producing space food, and highlights the imperative measures that need to be put into effect.

## Quality and safety of food

In general, the quality of food depends on various properties being safety, nutritional value and sensory attributes that determine their acceptability and, thus, consumption both on earth and in space^5^. Objective indicators and parameters determined by appropriate methodologies can evaluate the qualitative properties and the suitability of a food for consumption (i.e. microbial counts, presence and concentration of toxic contaminants, and nutritional value) or indicate the occurrence and development of undesired degradation processes and corresponding changes in the product that could impair their acceptability (e.g. lipid oxidation and rancidity with off-flavours).

From the inception of spaceflight, safety has remained a fundamental quality requirement for space food. This focus has guided the identification and choice of preservation methods, with the primary objective being the minimization of health risks and microbial threats. However, processing (i.e. type and applied parameters) could significantly affect the final quality of a food, independently on the initial quality of the raw material, formulation or recipe of the product. In general, intense processes such as those needed and used for space food manufacturing (i.e canning, drying, freeze-drying) while determining the desired safety can trigger degradation reactions that may impair both the nutritional value and the qualitative properties especially the sensory ones (e.g. flavour, taste and colour). Moreover, degradation reactions and phenomena could still occur even in microbiologically stable products and evolve during the mission duration especially in a medium- to long time storage time. These could lead to the formation of off-flavours due to rancidity and/or other oxidative reactions, change the physical properties of biomolecules (e.g. starch crystallisation and retrogradation), modify the structural and colloidal properties of the multiphasic food (e.g. emulsified food, e.g. sauces, juices) by impairing both the sensory acceptability (texture, flavour, aroma) and nutritional value. So far, limited information and studies have been carried out on the impact of the environmental conditions on the qualitative and safety properties of processed space foods and, in turn, on the corresponding effects on the sensory properties that can impair their acceptability especially considering long-term missions.

The availability of safe, high quality food with an estimated shelf-life (i.e. the length of time for which a product remains acceptable and fit for consumption) overlapping with that of the space missions is mandatory to guarantee an adequate (macro- and micro-) nutrients intake but also the willingness of space travellers to eat.

In recent times innovative and gentle techniques have been proposed as alternative to the conventional ones to produce long shelf-life space food with higher sensory and nutritional value including high hydrostatic pressure and its combination with high temperature (pressure assisted thermal sterilisation-PATS or High Pressure Thermal Processing-HPTP), microwave assisted thermal sterilization, novel dehydration technologies and food 3D printing^5–9^. Safety and extended shelf-life of foods is highly depending also on the packaging despite for space missions other aspects (e.g. mass, volume, time and waste disposal capacity) need to be considered in the development of materials with improved technological performances in terms of higher barrier abilities against environmental stresses in space (Evans, 2023).

While the microbial stability is compulsory and achieved by intense stabilisation processes, still scarcely investigated are:

- Individual and combined effect of mission’s duration (medium-, long-), microgravity, radiation on the degradation reactions and processes affecting quality and nutritional value of foods and their safety. This needs to be investigated with a systematic approach with main focus on: chemical or oxidative reactions (i.e. lipids) that may lead to the formation of off-flavours and toxic compounds and/or changes in sensory properties; spontaneous phenomena (e.g starch retrogradation in bakery products and intermediate moisture food), destabilisation of colloidal systems (e.g. emulsions and emulsified foods, e.g. beverages, liquid-semi-liquid foods).
- Optimisation of process conditions and food space formulations or recipes in order to limit and/or counteract the unavoidable degradation reactions (e.g. use of antioxidants, selection of ingredients) and quality loss.

## Sensory properties—senses and appetite

There is no objective evidence, but only anecdotal information, on how appetite and sensory (and, in particular, the olfactory ones) perception of foods are affected by spaceflight-induced stresses, environmental factors (e.g. microgravity, radiation, confinement, isolation) or factors connected with food consumption and/or preparation within the spacecraft (e.g. spacecraft background smell, CO_2_-concentration in the air, water quality, etc.)^2^. Appetite, intended as the feeling to eat food is the key element for any adequate nutrition in any healthy human. In this context, appetite is affected by physiological and psychological factors, food-related properties, which, in turn, could be impaired by microgravity or low-gravity conditions. Additionally, changes in neurophysiological and neuro-behavioural performances need to be considered. Foods with sensory properties that don’t match the expected quality due to the presence of off-flavours, unexpected texture or undesired colour become less attractive resulting in a reduced consumption; moreover, the majority of space foods are processed to an extent that they lose the “freshness” attributes of the corresponding raw ingredients and material aspect that can further limit the willingness of the astronauts to eat. As examples, consider canned vegetables which, as a result of the overcooking effect induced by the sterilisation process to ensure microbial safety, exhibit an excessive softness. Additionally, dried products may display differences in aroma, colour and texture compared to their fresh counterparts, due to the effect of the drying process. Post-mission interviews with astronauts on some of the longer recent space flights highlighted that one of the most common complaints is the lack of freshness in the food^10,11^. The concept of freshness is undoubtfully rather complex^12^, being used to refer to many different aspects of food/flavour experiences depending on the specific food under consideration (e.g. freshly-grown, freshly-ground, freshly-made, a fresh taste, etc.). In this frame, also the lack of noise at consumption (e.g. crunchiness) is related to a lack of freshness for some foods^12–14^ and the lack of sound during eating could, thus, influence food intake. Appetite is the result of physiological and psychological factors that intercept the feeling of eating based on the desired and perceived sensory properties of a food. Some recent evidences highlighted the main role of space-living conditions as well as the decrease of the qualitative properties of foods during space missions’ time. To limit malnutrition or undernutrition of space travellers as related to a lower appetite there is the need to investigate:

- Which are the main environmental factors (CO_2_, sound, water quality, etc.) impacting appetite regulation;
- The relative importance on appetite regulation of the physiological, psychological and neurophysiological factors and those related to the qualitative properties of space foods and their changes during the storage in the missions.

## Bioavailability and bioaccessibility

The nutritional and health status of astronauts and their performances do not depend only on the amount of food and related content of macro-, micro-nutrients, fluid and bioactives consumed but also by their bioaccessibility and bioavailability, i.e. the fraction of the ingested bio-component that reaches the systemic circulation (blood flow) to be distributed to organs and tissues where it manifest its bioactivity^15^. Bioavailability depends on the bioaccessibility of the biomolecule from the food, its absorption and transformation occurring during digestion and overall these processes depend on many food- and human-related factors (e.g. processing and formulation, qualitative properties of space food, gut microbiota, etc.)^16,17^. To increase the bioavailability recent scientific studies are highlighting the importance of the presence in foods either of components that act as “solvent” (i.e. lipids for hydrophobic bioactives) or colloidal structures (i.e. oil-in-water emulsions) whose properties can favour the absorption of the target bioactives. These concepts have been implemented in the design and formulation of “excipient foods”^18^.

New and innovative foods like the microbial ones (single-cell protein sources) and the nutrient-dense functional foods (e.g. kale, nuts, legumes, oil etc.) produced during space flights, as well as supplemented nutraceuticals and pharmabiotics still have to show scientific evidence to complement space food. They need, as well, to be assessed for both their contribution to the astronauts diet in terms of macro- and micronutrients, quality and safety and the bioaccessibility and bioavailability of nutrients and active compounds.

Bioavailability is an essential parameter for assessing the relationship between food and its health benefits^19^. There is a number of investigations ongoing to study the processes and mechanisms involved in the bioavailability of nutrients and bioactive but limited in the case of space food that highlight the specific needs of studies:

- Understanding the relationship between the changes of food space quality during space missions induced by degradative reactions and spontaneous physical processes and bioavailability in both quantitative and qualitative terms;
- Need and strategy for biofortification to increase the bioefficiency of bioactives of space foods while limiting the risks of toxicity;
- Design and development of excipient foods for space missions to increase the bioavailability of nutraceuticals and bioactives.

## Nutritional status

Although energy intake of space travellers on the International Space Station (ISS)—as well as during previous missions—up to today is estimated to be about 25 % below energy requirement, associated with insufficient supply of other nutrients^20^, there are still no tools to accurately measure energy expenditure in space missions. Consequences of insufficient energy supply are mobilization of endogenous fat and protein stores resulting in loss of muscle mass and strength and, besides others, weakening of the cardiovascular and immune system^20^. To ensure adequate energy and nutrient supply, both space agencies, NASA and ESA, started to monitor energy intake, but there is still missing information in the food database and not all nutrients and water content data are available for the food items included. Therefore, the assessment of energy, water or mineral balances of astronauts remains a challenge. On the other hand, nutrient supply and, in particular, undernutrition is known to affect vital physiological systems.

For instance, insufficient caloric consumption mobilizes fat mass as an energy store. Concomitantly to fat mass, however, protein will also be broken down to use amino acids as a fuel source. As a result, hypocaloric nutrition leads to lower lean body (muscle-) mass on top of the muscle degradation because of inactivity^21,22^. Semi-starvation also affects the cardiovascular system e.g. causing lower orthostatic tolerance (reviewed in ref. ^20^) and weakening the immune system by inflammation processes^23,24^. Motor and cognitive function might also be decreased by insufficient caloric intake and could affect the ability of space travellers to perform work-related tasks mandatory for landing. Based on studies carried out in military, where negative energy balance is common during military operations, total energy balance was associated with lower-body power and strength^25^. Therefore, astronauts who are semi-starving might be expected to have early decreases in endurance and later decreases in muscle strength in parallel to reduced muscle mass.

The current available space food on ISS is rather well-balanced with regard to nutrient composition and provides—if sufficient calories are consumed—adequate nutrient supply (reviewed in ref. ^20^). This is valid also for ISS recommended values for micronutrient (vitamins and minerals) intake that are mainly reached if the required caloric intake to maintain body mass is provided^26^. Consequently, hypocaloric (i.e insufficient caloric intake) nutrition and/or reduced food intake will not only lead to inadequate energy supply, but will also contribute to general malnutrition, including vitamin and mineral supply. This becomes relevant especially in long-duration missions (e.g. the exploration missions), where the bioavailability and bioaccessibility of bioactives might be modified or reduced due to unavoidable food quality changes.

**Fig. 3:**
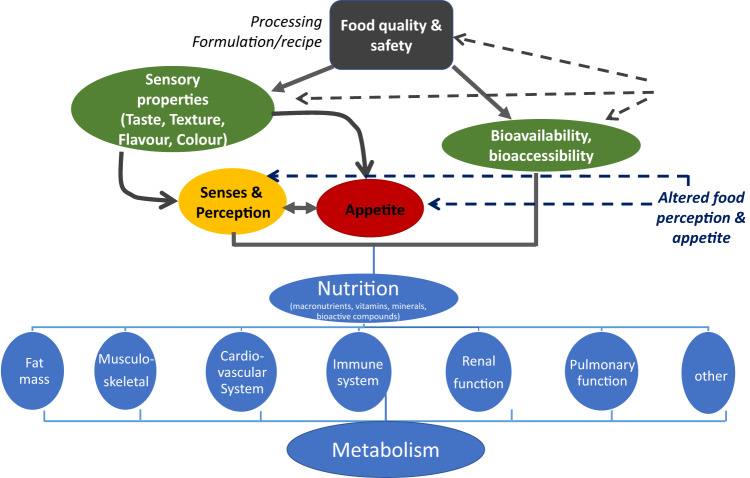
Food, nutrients and metabolism. Food related factors affecting nutritional supply and metabolic interactions.

Taken together and depicted in Fig. 3, all aspects of nutrition are expected to affect metabolism and a very complex analysis of the interactions with the physiological systems and thereby the health and performance during exploration missions, is needed (Fig. 3). Concomitantly, priorization of respective research questions is mandatory.

The main goal during exploration missions to keep space travellers healthy will be to maintain energy balance. However, there are several gaps, which need to be filled in different areas affecting energy balance. In the following paragraphs the respective research questions connected with the goal of maintaining energy balance and optimal nutrient supply are listed:

- Energy-/Nutrient-/Fluid availability

Up to now, there is no possibility to produce food onboard rockets/system flying space travellers to Moon or Mars or any other outer planet. On ISS there is a small greenhouse available, which, however, is used for research purposes and is not aimed to supply vegetables, lettuce or any other fruits in a sufficient amount to the crew. Further scientific and technological research is needed to provide sufficient amount of crops for space explorers on the way to or on Moon, Mars and beyond^27^. Right now, to be able to provide sufficient calories for the crew, rather ‘energy dense’ food needs to be produced, stored and consumed. Examples of ‘energy dense’ foods (i.e. high energy/calories in reduced weight or volume) are energy bars or nuts or oils, although no one has ventured to cultivate the latter in space thus far. Oils of different origin like the olive oil, flaxseed oil, or canola oil can even offer a substantial proportion of mono- and polyunsaturated fatty acids. However, it’s worth noting that food technology must come into play in this context, as the latter fats are less stable and can potentially lead to rancidity if they are not properly protected by naturally present (e.g. polyphenols and other bioactives in extra-virgin olive oil) or added antioxidants. Even when considering the transportation of energy-dense food, it becomes evident that this would impose a substantial weight and require a significant amount of space within the spacecraft. This raises a fundamental question: can we accept a certain level of caloric restriction on the crew to diminish the weight and volume burden, and if so, to what extent can we do so without compromising their cognitive and physical performance? Moreover, reducing caloric intake through food restriction not only results in a decrease in calories and macronutrients like protein, fat and carbohydrates, it also entails a reduction in micronutrients. Here, again, the question arises: higherto what extent and for what duration can astronauts endure a lowered intake of micronutrients, and what specific types of micronutrients are subject to reduction? Malnutrition of different micronutrients may lead to respective pathophysiological effects at different intake levels. This aspect thus, has to be examined on the respective individual level of all the micronutrients.

Provision of nutrients not only depends on the amount provided with the diet, being their main impact is affected by bioavailability and bioaccessibility. Another way forward therefore could be, to increase bioavailability and bioaccessibility. For example, if usually the gastrointestinal tract absorbs 30% of a particular nutrient from a specific product, advancements in product manufacturing technology (e.g. encapsulation) or modifications of the formulation in the production process could be optimised to enhance absorption rates. Consequently, this would result in higher levels of that nutrient being assimilated by the body. The questions are: what are the technological improvements leading to higher bioavailability and -accessibility of respective nutrients? Which technological solutions (including selection of ingredients and formulation and/or processes) could be used to improve them?

- Exercise prescription

Intense research has gone into the development of effective exercise protocols to prevent muscle and bone loss in space. Since the problem still persists, the success is still questionable^28^. The latter review by Peter Stein^28^ names three contributing factors to muscle and bone loss in microgravity: the reduction in remodelling, the pre-flight fitness level and the inability to maintain energy balance during the missions. To maintain energy balance during the mission the question is what is the main driver of total energy expenditure in spaceflight? Is it a change in resting metabolic rate, or is the main cause physical- and non-physical activity within the respective timeframe? Resting metabolic rate is the energy expenditure a mammalian metabolism needs during a period of strict and steady equilibrium conditions^29^. Based on the results published by Bourdier et al.^30^. examining changes in energy expenditure during ISS-missions, it seems that resting metabolic rate is not much affected in microgravity, but physical and non-physical activity seem to be to the main driver for changes in total energy balance in microgravity. A daily regimen of physical activity is recommended on ISS, comprising two hours of endurance and strength training, including the setup of the equipment. This results in about 25 min of aerobic and 30 min of resistive training per day^30,31^. A daily routine of physical activity of two hours of endurance and strength training is advised. However, when considering energy expenditure, resistive exercise has been found to have lower energy costs compared to aerobic exercise^32^. On the other hand, high-intensity interval training conducted using a hydraulic resistance system appears to offer the advantages of both resistance and endurance training while requiring less time^33^. For future exploration missions it is therefore crucial to optimize exercise routines and energy expenditure to preserve cardiovascular and musculoskeletal health without exceeding the necessary energy expenditure to maintain energy balance and placing excessive stress on payload weight.

In combination of the not yet found optimal exercise countermeasure and the energy needs for exercise the question remains, what is, in general, the optimal and personalized exercise prescription that minimize negative energy balance and maintains performance? Mission duration or time in mission might also lead to different exercise needs and here we also need to ask what is the optimal personalized exercise prescription that minimize negative energy balance and maintains performance in the time course of the mission? In summary, we need to find the optimal exercise and non-exercise prescription with the lowest energy expenditure level.

- Technology development

Measuring total energy expenditure, resting metabolic rate or activity and non-activity related energy expenditure is not common in space missions. Tools which are available on Earth are not available on ISS. The complex ENERGY experiment carried out by Stephane Blanc and his team^30^ used all the actually available measures to determine the different aspects of energy expenditure during 6-month missions on the ISS. However, to provide optimal energy supply to the crew it is mandatory to provide or develop respective technology for real-time assessment of energy expenditure. Here we need to examine which of the shelf devices (biologgers), grouped together, may provide quantitative/objective, real-time information on total energy expenditure (basal metabolic rate, physical activity induced energy expenditure) and body composition? Smart (validated) devices are needed which provide real-time information on body temperature, daylight, heart rate and kind of physical activity? Finally, the question is, is there hardware/software available, which measures real-time nutrient intake with an adequate accuracy?

- Further countermeasure ideas

The composition and functionality of the microbiome most likely changes during spaceflight^34,35^. Supporting a healthy microbiome by respective measures in space travellers might maintain their health during the mission but also support rehabilitation when being back on Earth. There is convincing data from clinical studies on ground using pre-, pro- and synbiotics showing that these can improve nutrient bioavailability^36–40^ and various aspects of health and physical performance (muscle mass and –strength, glucose tolerance, inflammation, immune system, cardiovascular health)^41–47^. Up to now, there is insufficient data to generalize these results to the general population. However, because pre-, pro- and synbiotics would be easy to implement and would be cheap countermeasures without adding additional upload mass by optimising their inclusion in the space food, their supplementation are proposed as a nutritional countermeasure for exploration missions.

- Metabolism

Metabolic inflexibility has been demonstrated in bed rest^48,49^ but no data is available from the ISS. However, insulin resistance was observed in space flight^31^. Metabolic inflexibility could be of concern for long-duration missions, but insufficient data is available to conclude whether it might be harmful in exploration missions. To get further insight into the danger of metabolic inflexibility during long-duration exploration missions the severity of metabolic inflexibility occurring during actual space flight needs to be explored as well as whether this metabolic inflexibility may reach medical concerns during exploration missions. As discussed above, the optimal relation between exercise and energy expenditure needs to be explored and within that area it is important to determine the relationship between metabolic flexibility/inflexibility and the existing applied exercise countermeasures together with the unknown underlying mechanisms.

## Conclusion

To be able to maintain energy balance in exploration missions and provide palatable and nutritious food in adequate amounts for space travellers future studies are needed to investigate respective technological solutions and tools to measure accurately and online total energy expenditure, resting metabolic rate and physical activity and non-physical activity derived energy expenditure as well as body composition. Research should be directed into (a) investigating off-the-shelf devices that, when combined, can offer real-time, quantitative, and objective data on various aspects of total energy expenditure and body composition, (b) exploring validated smart devices that are currently accessible and capable of providing real-time data on parameters such as body temperature, daylight exposure, heart rate, and the type of physical activity being performed and (c) examining existing hardware and software solutions that can accurately measure real-time nutrient intake. Technology and protocols are also to be researched to provide palatable food maintaining or even increasing bioavailability and -accessibility. Optimal exercise regimes in relation to energy expenditure need to be developed to maintain muscle and fat mass which, concomitantly, do not burn more calories than needed.
